# Ergonomics and musculoskeletal disorders in neurosurgery: a systematic review

**DOI:** 10.1007/s00701-020-04494-4

**Published:** 2020-07-23

**Authors:** Alexandre Lavé, Renato Gondar, Andreas K. Demetriades, Torstein R. Meling

**Affiliations:** 1 Department of Clinical Neurosciences, Division of Neurosurgery, Geneva University Hospitals, Geneva, Switzerland; 2 Department of Neurosurgery, Western General Hospital, Edinburgh, UK; 3 Faculty of Medicine, University of Geneva, Geneva, Switzerland

**Keywords:** Surgery, Systematic review, Ergonomics, Neurosurgical practice, Spine surgery, Neuro-endoscopy, Musculoskeletal disorders

## Abstract

**Background:**

Work-related musculoskeletal disorders (WMSDs) are a growing and probably undervalued concern for neurosurgeons and spine surgeons, as they can impact their quality of life and career length. This systematic review aims to ascertain this association and to search for preventive measures.

**Methods:**

We conducted a PRISMA-P-based review on ergonomics and WMSDs in neurosurgery over the last 15 years. Twelve original articles were included, of which 6 focused on spine surgery ergonomics, 5 cranio-facial surgery (mainly endoscopic), and one on both domains.

**Results:**

We found a huge methodological and content diversity among studies with 5 surveys, 3 cross-sectional studies, 2 retrospective cohorts, and 2 technical notes. Spine surgeons have sustained neck flexion and neglect their posture during surgery. In a survey, low back pain was found in 62% of surgeons, 31% of them with a diagnosed lumbar disc herniation, and 23% of surgery rate. Pain in the neck (59%), shoulder (49%), finger (31%), and wrist (25%) are more frequent than in the general population. Carpal tunnel syndrome showed a linear relationship with increasing cumulative hours of spine surgery practice. Among cranial procedures, endoscopy was also significantly related to shoulder pain while pineal region surgery received some attempts to optimize ergonomics.

**Conclusions:**

Ergonomics in neurosurgery remains underreported and lack attention from surgeons and authorities. Improvements shall target postural ergonomics, equipment design, weekly schedule adaptation, and exercise.

## Introduction

Neurosurgeons and spine surgeons are exposed to work-related musculoskeletal disorders (WMSDs), which can negatively impact their quality of life and career length. According to the World Health Organization definition, WMSDs include all health problems of the locomotor apparatus (the skeleton plus muscles, tendons, cartilage, ligaments, and nerves) and all relevant forms of ill health, ranging from mild or transitory disorders to irreversible and disabling injuries [31].

Spine neurosurgeons in particular are exposed to WMSDs by adopting sustained non-neutral positions, with prolonged neck flexion and coronal misalignment as they operate in a standing position, frequently leaning over the operating table [19]. A significant prevalence of neck and back pain has been reported among spine surgeons [2]. The use of vibrating power tools, Kerrison punches with repetitive hand movements, lead aprons, and high surgical loads pose risks for repetitive strain injury, carpal tunnel syndrome, and wrist or hand dysfunction. Other examples of preventable disorders affecting muscles, tendons, and nerves include tendinitis, degenerative spine diseases, thoracic outlet syndrome, and tension neck syndrome.

WMSDs may lead to time off work and could negatively impact the quality of a surgeon’s performance [14]. An increasing number of studies have drawn attention to the importance of postural ergonomics, aiming to reduce musculoskeletal fatigue among neurosurgeons [1].

We conducted a systematic review and meta-analysis of published studies on this topic. Our goals were to determine (1) the prevalence of WMSDs in neurosurgery and spine surgery, (2) their burden on quality of life and ability to work, and (3) the possible preventive interventions to minimize their consequences.

## Material and methods

### Search strategy and study selection

According to the PRISMA-P guidelines (Preferred Reporting Items for Systematic review and Meta-Analysis Protocols checklist) [26], the authors systematically reviewed the published literature on ergonomics and musculoskeletal disorders in neurosurgery.

The literature search was performed on MEDLINE (US National Library of Medicine), Embase Library, and Google Scholar, including papers from 2006 to 2020. The following Medical Subject Headings (MeSH) terms were used: “ergonomics”[MeSH Terms] OR “ergonomics”[All Fields]) AND (“neurosurgical procedures”[MeSH Terms] OR (“neurosurgical”[All Fields] AND “procedures”[All Fields]) OR “neurosurgical procedures”[All Fields] OR “neurosurgery”[All Fields] OR “neurosurgery”[MeSH Terms]”.

**Fig. 1:**
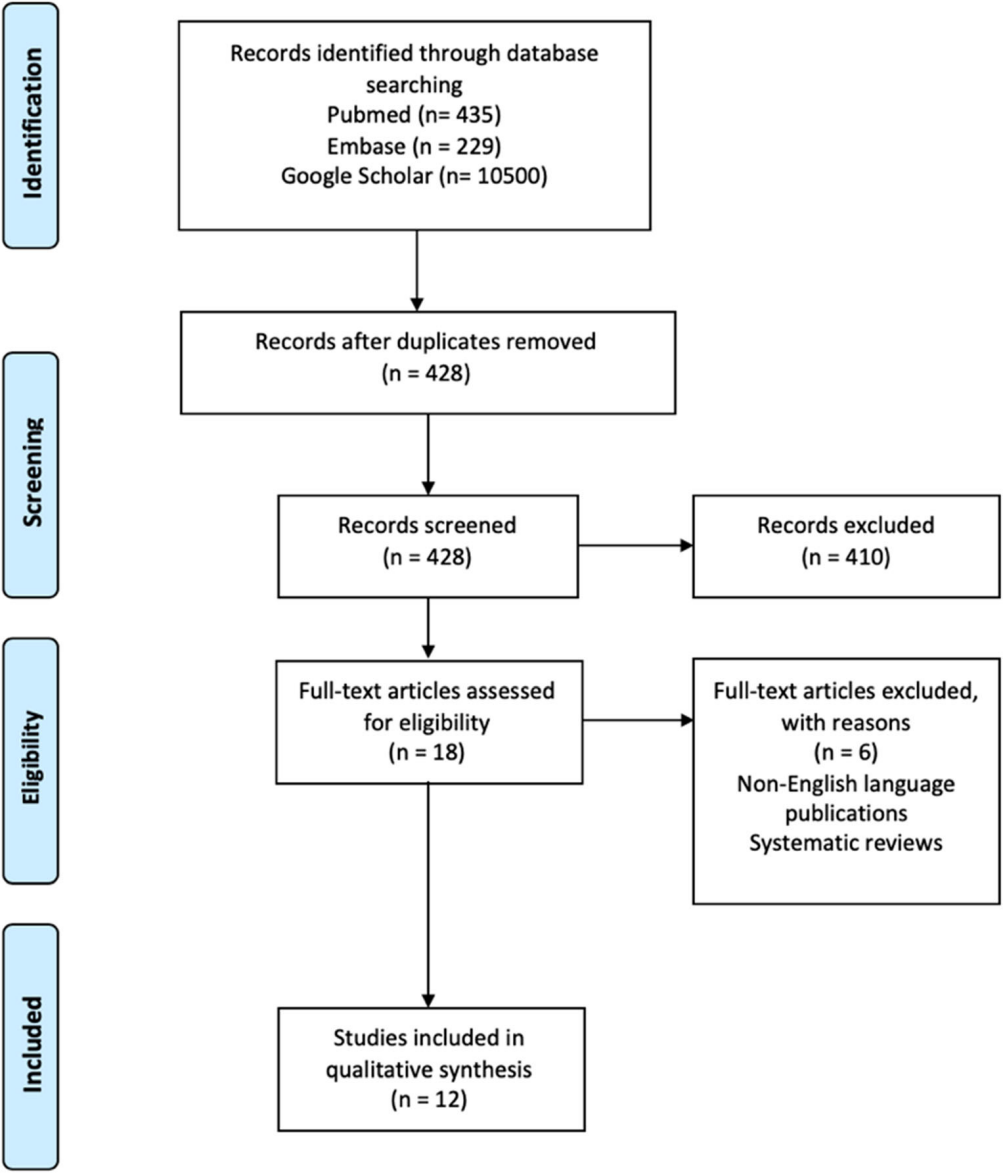
PRISMA flowchart

No limits were defined regarding the year of publication, language, or publication status, and no other additional filters were applied after running the search in the abovementioned databases (Fig. 1).

After removing duplicates, the titles and abstracts from 428 articles were screened. Two authors (AL and RG) independently screened titles and abstracts of all identified articles, and full-text copies of all relevant articles were acquired.

The following inclusion criteria were used: (1) peer-reviewed research articles, prospective, or retrospective, on ergonomics in the field of neurosurgery and spine surgery and (2) studies written in the English language.

### Risk of bias and quality of study

The accepted articles were independently graded by two authors (AL and RG) according to the Newcastle–Ottawa Quality Assessment Scale [30] for quality assessment of non-randomized studies. The level of evidence for each study was evaluated using the Oxford Centre for Evidence-Based Medicine guidelines.

### Data collection

The following parameters were extracted and registered for each article: (1) study ID; (2) study characteristics (author, year, country, prospective, or retrospective study); (3) patient demographics; (4) sample size; (5) ergonomics assessment; (6) type of surgery performed; (7) outcome measurements; (8) follow-up (FU) time. If necessary, the consensus was reached by both authors through discussions with the senior author (TRM). Data from each study reporting prevalence estimates were extracted.

### Statistical analysis

Results for continuous variables are reported as mean ± standard deviation (SD) or range. For articles that did not report mean and SD, we estimated the mean and SD according to the methodology described by Hozo et al. [15]. Categorical variables are presented as median and quartiles or by absolute and relative frequencies.

## Results

In total, 18 titles were retained for full-paper screening. Six papers were excluded from the qualitative analysis as these were systematic reviews or not written in English. Thus, twelve studies were included in the final analysis (Fig. 1).

Six articles [1, 2, 12, 17, 19, 20] focused on spine surgery ergonomics and related issues; five others discussed cranio-facial surgery [5, 9, 22, 23, 28], mainly endoscopic, and one survey reported on both spinal and cranial surgery [14].

It was not possible to aggregate demographic results as the variability of study designs and purposes was significant. Among the spine-oriented studies, three were surveys, three cross-sectional studies (randomized or not), and one was a technical note. As for the cranio-facial-oriented studies, these comprised two surveys, two retrospective descriptive cohorts, and one technical note. Among these last ones, only one included microscopic approaches, while the rest focused on endoscopic sinus and skull base surgery. Due to the diversity of parameters measured and questions reported, no quantitative analysis was performed. The results are shown in Table 1**.**

**Table 1:** List of the clinical data available from the 12 articles included in the review

Source	Year of publication	Design	Specialty	Geographic location	Study participant	Principal outcomes
Gadjradj et al. [14]	2020	Survey	Cranio-facial and spine	The Netherlands	*N* = 417	- 73.6% experienced WMSDs. - Spine surgery as first cause of WMSDs. - 11.3% of the respondents had to take time off work.
Auerbach et al. [2]	2011	Survey	Spine	USA	*N* = 561	- 38% of respondents experienced neck pain/strain/spasm. - 31% of respondents reported lumbar disc herniation/radiculopathy. - 28% cases of cervical disc herniation/radiculopathy. - 24% of rotator cuff disease among the respondents. - 20% reported varicose veins or peripheral edema - 18% experienced lateral epicondylitis.
Forst et al. [12]	2006	Survey	Spine	USA	*N* = 371	- 107 reported cases (28.8%) of CTS. - Average latency from beginning of residency to the development of CTS: 17.1 years. - Neurosurgeons as a subgroup showed a significant increase in risk of CTS (adjusted OR = 2.03). - Kerrison rongeur was the greatest occupational risk to the upper extremities (adjusted OR = 2.72). - 30.8% of the surgeons with CTS reported that the chronic pain interferes with their work, and 23.1% modify the way they perform surgery because of CTS.
Park et al. [19]	2012	Randomized crossover	Spine	South Korea	*N* = 12	- Lumbar lordosis, cervical lordosis, and occipital angle closer to natural standing values when discectomy was performed with a loupe. - Whole spine angles closer to natural standing position with table height at midpoint between the umbilicus and sternum.
Park et al. [20]	2013	Randomized crossover	Spine	South Korea	*N* = 18	- Whole spine angles closer to natural standing position closer to natural standing values when discectomy was performed with a microscope. - When using a microscope, lumbar lordosis, thoracic kyphosis, and cervical lordosis showed no differences according to table heights above the umbilicus.
Van Lindert et al. [28]	2004	Retrospective	Cranio-facial	The Netherlands	*N* = 269	- HMD decreased visual strain. - Working position was considered to be more comfortable when wearing the HMD. - Eye-hand coordination was improved thanks to HMD.
Choque-Velasquez et al. [5]	2018	Retrospective	Cranio-facial	Finland/Italy	N = 56	- Steeper sitting position with the upper torso and the head of the patient bent forward and downward allows a more ergonomic working position for the neurosurgeon allowing his/her arms to rest over the patient’s shoulders during the procedure.
Albayrak et al. [1]	2007	Cross-sectional	Spine	The Netherlands	*N* = 7	- EMG results showed an average reduction of 44% for the erector spinae muscle, 20% for the semitendinosus muscle, and 74% for the gastrocnemius muscle. - Support considered as comfortable, safe, and simple to use
Ramakrishnan et al. [22]	2019	Survey	Cranio-facial	USA	*N* = 250	- Operating in the standing position resulted in physical discomfort mostly of the trunk and lower body. - Operating in the sitting position shifted the muscular strain to the upper back, neck, and shoulders.
Ekanayake et al. [9]	2017	Technical note	Cranio-facial	UK	*N* = 350	- Semi-sitting position, with the addition of having the patient’s head flexed and turned to the side of the surgeon allowed a more comfortable and intuitive placement of the operator’s arms in relation to the nares.
Ramakrishnan et al. [23]	2017	Survey	Cranio-facial	UK	-	- Discomfort after endoscopic procedure in the standing position was worse in the legs and low back, whereas, in the sitting position, it was predominant in the upper back and arms.
Ito et al. [17]	2015	Technical note	Spine	Japan	*N* = 14	- Simulations performed using the support significantly reduced the musculoskeletal loading on the ventral side of the left foot by 70%. - The device generated circa 10% of the musculoskeletal load on the right hand.

## Discussion

The current review allowed us to summarize the state-of-affairs in a growing field that has been neglected for many years; to our knowledge, this is the first of its kind in neurosurgery. Firstly, ergonomics in neurosurgery remains underreported and lack attention from surgeons, hospital administrations, surgical material designers, and health insurance companies. Secondly, the challenges in ergonomics are quite different between spine surgery and cranio-facial surgery as the volume of pathology, as well as the instruments and positions used, are substantially different. Thirdly, the impact of such problems on surgical performance and surgeons’ longevity and quality of life is largely unknown. Lastly, any formal attention given to such aspects during the formative years of neurosurgical training seems non-existent.

Like other groups working in medical health, our review shows that surgeons are significantly impacted by musculoskeletal disorders. According to the statistics of the Health and Safety Executive, the UK government’s agency responsible for the encouragement, regulation, and enforcement of workplace health, there were 60,000 work-related cases of the musculoskeletal disorder among people working in human health and social work in Great Britain in 2019 [16]. This rate is significantly higher than for workers across all other industrial branches. Surgeons are not spared from musculoskeletal disorders and are even considered at high risk of developing WMSDs [3, 10].

Among spine surgeons, sustained neck flexion is seen as a major risk factor for musculoskeletal disorders [14, 19, 20]. Such posture is frequently necessary during procedures, particularly during cervical spine approaches. An additional contributing factor may be that surgeons tend to neglect their posture during surgery as the procedure itself requires full concentration.

Auerbach et al. [2] surveyed the type and prevalence of musculoskeletal disorders among 561 surgeon members of the Scoliosis Research Society; the majority of complaints were low back pain (62%), neck pain (59%), and shoulder pain (49%). A significant number of surgeons suffered also from leg pain with radiculopathy (31%), finger pain (31%), neck pain with radiculopathy (28%), elbow pain (28%), and wrist pain (25%) [2]. The prevalence of neck pain in the general population is approximately 20% [25]. Another statistic from this study is particularly insightful: 4.6% of the respondents had undergone surgical intervention for cervical disc disease. This is in major contrast to the prevalence of 0.35% of cervical radiculopathy observed in the general population [13] and this difference was attributed by some authors [2] to the position with the neck flexed during extended time periods. Importantly, 31% of the surgeons in this survey reported lumbar disc herniation and symptomatic back pain with radiculopathy, with a surgery rate of 23% [2]. Here, again, there is a gap between the prevalence of symptomatic lumbar herniated disc in the general population (up to 5% [29]) and the prevalence observed among the 561 surgeons surveyed. Prolonged operative times while standing, awkward spine position and movement, or lifting heavy patients have been implicated to explain such a difference.

Similar findings were reported by Gadjradj et al. in a survey among members of the Congress of Neurological Surgeons [14]. Neck, shoulder, and back complaints were the most prevalent in the context of three procedures: lumbar discectomy, ventriculo-peritoneal shunting, and endoscopic third ventriculostomy (ETV). Neck complaints were the most prevalent in respondents performing lumbar discectomy. Again, surgical procedures for degenerative spinal diseases were most frequently mentioned as preceding factors before such complaints. Univariate analysis showed that tenure of less than 15 years and an operating room (OR) furnished ergonomically were associated with fewer WMSDs. Multivariate analysis, however, only revealed that having a tenure of less than 15 years was protective. Finally, height, sex, age, dominant hand, caseload, the average duration of the procedure, hours per week in the OR, and having the OR furnished ergonomically were all non-significant factors.

A high rate of carpal tunnel syndrome (CTS) among spine surgeons was reported by Forst et al. [12]. The survey included 371 respondents including 274 spine specialists, where 16.4% were neurosurgeons and 57.4% were orthopedic surgeons. When compared with a group of non-surgeons, the odds of developing CTS were at least twice as high for spine surgeons as for non-surgical medical practitioners, with 29% of the surgeons reporting CTS [12]. The risk of developing CTS showed a linear relationship with increasing cumulative hours of surgery. A particularly relevant observation for neurosurgical practice is that the use of the Kerrison rongeur appeared to be a major risk factor for the development of CTS. Indeed, the neurosurgeon subgroup showed a significant increase in the risk of developing CTS, with an adjusted odds ratio of 2 [12]. Other significant predictors of CTS among all medical personnel were obesity and a length of professional practice of more than 5 years. Auerbach et al. [2] reported a lower rate of CTS of approximately 9%. Some discrepancies in the rate of CTS observed between Forst et al. [12] and Auerbach et al. [2] could be explained by the potential response bias in the survey of Forst et al. [12], with a low response rate of 16.8%.

According to Gadjradj et al. [14], ETV is associated with a high rate of muscular complaints. In the literature, endoscopic and laparoscopic procedures have also been associated with high rates of WMSDs [11, 22–24, 27]. Neurosurgeons are not performing laparoscopic procedures, but endoscopic surgery in a standing position is frequent in neurosurgical practice. In particular, upper limb complaints are frequently reported [2, 14]. Forty-nine percent of surgeons in the survey by Auerbach et al. [2] suffered from shoulder pain, compared with a lower percentage at 24.7% in Gadjradj et al. [14]. Interestingly, 100% of the surgeons surveyed in the latter reported shoulder pain while performing ETVs.

In an online survey among members of the European Rhinologic Society [24], 80% of the 250 responders reported musculoskeletal problems. Neck and back were the commonest sites of symptoms, in approximately 60% of cases. A positive correlation was found between musculoskeletal symptoms and the standing position. Ramakrishnan et al. [23] compared the sitting and standing positions in a study on endoscopic sinus surgery and showed that the standing position led to increased discomfort in the lower extremities and back compared with the sitting position, which caused more discomfort in the upper extremities. Hand, neck, and eye discomfort were reported in both conditions. Rather than reducing muscle strain, the authors pointed out that the positional switch only transferred the tension to other muscle groups. Thus, there is still some controversy as to whether a sitting or standing position offers the most optimal ergonomic position during endoscopic sinus surgery.

As described above, lengthy hyperflexion of the cervical spine is associated with musculoskeletal disorders. To determine the optimal sagittal balance of the spine during neurosurgical procedures, Park et al. [19, 20] compared whole spine angles of 12 spine surgeons simulating surgeries on spine models. Three different methods to visualize the surgical field (naked eye, loupe, and out of loupe) were used and three different operating table heights were suggested. The authors showed that appropriate operating table height was the first effective step to improve ergonomics in the OR. Utilization of loupes should be coupled with a table height situated at the midpoint between the umbilicus and sternum and is comparable with a natural standing position in terms of values of lumbar lordosis, thoracic kyphosis, cervical lordosis, and occipital angle. Moreover, all parameters were also close to the natural standing position when a microscope was used during the simulation [20] if an optimal height of the operating table (midpoint between the umbilicus and sternum) is provided. As recently pointed out by Demetriades et al., microsurgery with a microscope is ergonomically more efficient than with loupes and headgear, at least in neurosurgery [8].

In order to improve ergonomics in neurosurgery, especially during spinal procedures that can be long and tiring, engineers at the Delft University of Technology developed ergonomic body support that supports surgeons during both open and minimally invasive procedures [1]. The aim was to reduce a surgeon’s muscle activity in the lower back and extremities while keeping the surgical field unobstructed. EMG results from seven independent participating surgeons showed that the prototype was effective in reducing the activity of the lower back and leg muscles during open surgery. This prototype, despite being described by some of the surgeons as restrictive to their movements, was considered safe in use [1]. Similarly, Ito et al. [17] proposed a body support device to reduce musculoskeletal loading for surgeons performing neurosurgical microsurgical procedures. This ergonomic device, tested on 14 neurosurgeons, efficiently improved the stability and smoothness of the surgeon’s motion while reducing musculoskeletal loading during procedures performed in the standing position [17].

Ramakrishnan et al. [22] recommended some simple measures to decrease WMSDs among surgeons performing skull base or endoscopic procedures. Appropriate monitor placement at 80–120 cm in front of the surgeon, as well as a correct adjustment of the table to keep the hand in line with the elbow at approximately 10 cm, with the arms slightly abducted and internally rotated could efficiently decrease the physical discomfort. As described earlier, the sitting position did not show a significant advantage in reducing muscular strain. The authors also cautioned surgeons not to look directly through an endoscope lens, to limit the time wearing headlight or loupes, and to limit wrist flexion, deviation, and rotation to less than 15 degrees [22].

Surgery of the pineal region is considered as a long and tiring procedure. In this context, some authors [5] proposed a variant of the classic position (semi-sitting or sitting position) to improve ergonomics, while reducing feared complications like air embolism [21]. Thus, they proposed a “praying position,” a position in which the patient is sitting steeper, with the head bent forward and downward and tilted about 30° [5]. Ergonomic modifications in patient positioning were also proposed by some authors [9] during endoscopic pituitary surgeries to improve operating by setting up conditions more comfortable for the neurosurgeon [9].

Lastly, head-mounted display (HMD) utilization during neuro-endoscopic procedures (mainly ETV and transsphenoidal pituitary surgery) was assessed in the retrospective study of van Lindert et al. [28]. This seemed to improve the ergonomics of neuro-endoscopic and other endoscope-controlled procedures by allowing the surgeon to have a neutral head position, regardless of the surgeon’s position relative to the surgical field. The authors showed that visual strain and ocular fatigue was decreased [28].

### Future directions

Trainees and young neurosurgeons must be aware of the significant risk of suffering from WMSDs and learn good practice early, as well as develop an awareness of the risk they are exposed to (for instance, the long-term use of the Kerrison rongeurs or loupes). Exercise also seems to reduce the risk of developing WMSDs in other standing and manual professions [7, 18, 32]. For example, some companies have set up workplace fitness programs, consisting of stretching and warm-up exercises for over-taxed joints. In the literature, exercise seems to have a moderate, but the definite impact, on WMSDs [31].

Challenges in ergonomics are quite different between the spine and cranio-facial surgery. Globally, fewer musculoskeletal disorders among cranio-facial surgeries specialist are reported, and most of the complaints are related to upper limb disorders [14, 23, 28]. Thus, ameliorations of ergonomics in cranio-facial surgeries are dependent upon correct patient positioning, utilization of HMDs, and correct positioning of the endoscope screen. Interestingly, neither of the sitting or standing position seems to show any advantage to reduce global muscular strain. In spine surgeries, improvements in postural ergonomic can be achieved by ideal positioning of the OR table [19, 20], the ideal positioning of the patient [5], the use of a microscope during spine surgeries [19, 20], and the use of body support to decrease surgeon’s muscle strain [1, 17].

Neurosurgeons may have to take time off work because of these WMSDs, and their surgical skills could also be altered by these. Specific costs generated from these forced vacations are not known, but overall, the National Research Council estimated that workers’ compensation costs associated with lost workdays due to musculoskeletal disorders range from $13 to $20 billion annually [6]. However, indirect costs must be added to the cost of compensation claims, leading to estimates as high as $45 to $54 billion annually for musculoskeletal disorders reported as work related [6].

As ergonomics is defined as the scientific study of people and their working conditions, especially done in order to improve effectiveness [4], it is a field that deserves investment, both in knowledge and in practice. It is evident that we still need to define optimal ergonomics for our specialty, paying due attention to (i) postural ergonomics; (ii) instrument design and technology; (iii) the environment around the surgeon (e.g., operating table, chairs, lights, monitors, navigation equipment); (iv) composition of operating lists (length of procedure, posture during each, weekly schedule); (v) exercise to avoid risk factors; and (vi) maintenance of good practice. Continuous self-improvement and lifelong learning are becoming essential components of a competent and successful surgeon.

### Limitations

The small amount of literature available on a specific topic is a natural limitation. The risk of selection bias was minimized by conducting a two-author data collection, as per PRISMA guidelines. Due to the diversity of parameters measured and questions reported, no quantitative analysis was performed. Lastly, there was not enough evidence synthesized in order to propose recommendations, other than further research in this area.

## Conclusions

Ergonomics for neurosurgeons has seemingly been neglected for many years and remains underreported. Surgeons, hospital administrations, surgical material designers, and health insurance schemes have a role to play in taking action to protect surgeons from this potential burden and occupational health hazard.

The impact of WMSDs in surgical performance and surgeon’s longevity and quality of life is largely unknown.

A systematic approach into the field of ergonomics in neurosurgery will require a sustained effort with multi-disciplinary input aiming at eventual long-lasting benefits for both neuro and spine surgeons, trainees, and patients.

## Abbreviations

WMSDs: Work-related musculoskeletal disorders
WHO: World Health Organization
CNS: Congress of Neurosurgical Surgeons
ETV: Endoscopic third ventriculostomy
OR: Operating room
CTS: Carpal tunnel syndrome
HMD: Head-mounted display
